# Vaccine equity: A fundamental imperative in the fight against COVID-19

**DOI:** 10.1371/journal.pmed.1003948

**Published:** 2022-02-22

**Authors:** The PLOS Medicine Editors

**Affiliations:** Public Library of Science, San Francisco, California, United States of America and Cambridge, United Kingdom

On March 11, 2020, WHO declared the outbreak of severe acute respiratory syndrome coronavirus 2 (SARS-CoV-2) a global pandemic. Now, almost 2 years on, COVID-19 continues to cause widespread morbidity, mortality, and disruption, both directly and indirectly, on a global scale. The speed at which multiple effective vaccines were developed is a remarkable achievement and testament to scientific advances and collaboration. However, numerous barriers to global vaccination efforts have left 47% of the world’s population unvaccinated or only partially vaccinated to date, with huge disparities between countries in the proportion of fully vaccinated individuals ranging from 0% to 95% [1]. Barriers such as vaccine hesitancy and anti-vaccine movements have hindered the progress of vaccination efforts, and have been perpetuated by fears over vaccine safety and the spread of misinformation and disinformation, despite the wealth of evidence supporting the benefits of vaccination. Adding to the evidence on vaccine safety, in this issue of *PLOS Medicine*, William Whiteley [2] and Steven Kerr [3] and respective colleagues have shown in large-scale observational studies that the Oxford-AstraZeneca vaccine is associated with no more than a small elevated risk of intracranial venous thrombosis and cerebral venous sinus thrombosis, respectively. The risks of cerebral venous thromboses are far greater following COVID-19 infection [4], further underlining the demonstrated benefits of vaccination.

Inequity of access to vaccines has posed a significant barrier to vaccination in low- and middle-income countries (LMICs), despite calls for action to achieve equitable distribution and production of COVID vaccines from WHO [5] and the UN Development Programme [6]. In addition to the health risks to unvaccinated individuals of contracting COVID-19, greater opportunities for infections and viral mutations [7] leave the world vulnerable to the emergence of new variants which threaten to evade our defences and undo progress made. Most recently, this has been seen in the emergence of the Omicron variant of concern. It is without doubt that vaccination rollout must be equitable and fair on a global scale. Despite tireless efforts by public health experts to extol the benefits of vaccine equity throughout the pandemic, global vaccination rates remain woefully unequal. As of February 1, 2022, approximately 183 COVID-19 vaccine doses had been administered per 100 people in high-income countries, compared to just 14 doses per 100 people in LMICs [8]. The COVID-19 Vaccines Global Access (COVAX) initiative was launched in April 2020 with the intention of addressing this imbalance through accelerated development, production and equitable distribution of vaccines. Yet, by December 30, 2021, only 7 African countries had achieved their target 40% vaccination rates [9], which leaves us with the question of how vaccine inequity can be tackled and what can be done to overcome barriers to vaccination.

To begin untangling this complex issue, we must first consider what a country needs to successfully vaccinate its population. A reliable supply of vaccines is the first step. The COVID Global Accountability Platform (COVID GAP) reported that in November 2021, just 20% of the doses pledged by G7 countries had been shipped to LMICs and there are additional reports of vaccines arriving close to their expiration dates, rendering them unusable [10]. Equally essential to vaccine rollout are health infrastructure, trained medical personnel, appropriate vaccine storage facilities, accessible vaccination sites, health literacy, and public willingness to take vaccines. Furthermore, limited supplies of vital equipment such as syringes risk derailing vaccination efforts [11], with shortfalls of between one and two billion syringes projected by the end of 2022 [12]. Scientists, academics and public health experts have collaborated to publish open letters to governments in high-income countries, recommending increased financial and operational support, and a temporary waiver of intellectual property rules to expand capacity for vaccine manufacture in LMICs themselves [13,14]. Of particular relevance is the World Trade Organisation (WTO) Agreement on Trade-Related Aspects of Intellectual Property Rights (TRIPS), which sets the minimum standards for regulation of different forms of intellectual property applicable to WTO member nations. In May 2021, delegations from WTO members representing multiple LMICs issued a communication proposing a waiver from certain provisions of the TRIPS agreement [15] to facilitate ‘the prevention, containment and treatment of COVID-19’. At the time of writing, a decision regarding the proposal is yet to be reached.

Expanding vaccine manufacturing capacity in LMICs offers an opportunity to bring the current pandemic under control and to enable a more coordinated, rapid global response to the current and future pandemics. Currently, Africa imports 99% of its vaccines [16], but the Africa Centre for Disease Control and Prevention (Africa CDC) launched the Partnership for African Vaccine Manufacturing in April 2021, with ‘the proposed ambition to manufacture 60% of Africa’s routine immunisation needs on the continent by 2040’ [17]. With this independence comes the potential to tailor vaccines to the needs of local populations, such as in outbreak situations, and to maintain the efficacy of vaccines through improved management of the vaccine cold chain. Such an ambition will only be possible with international cooperation, including from the pharmaceutical companies that own the intellectual rights to the vaccines. Progress is being made towards increasing production of COVID-19 vaccines in Africa [18,19]; however, the projected annual manufacturing rates fall short of meeting the needs of the continent’s 1.3 billion inhabitants [16], particularly when factoring in the multi-dose regimen for COVID vaccines. Given that 120 pharmaceutical companies have been identified as meeting the technical and quality standards required for manufacturing sterile injectables across Asia, Africa and South America [20], there is significant potential for introducing geographic diversity in vaccine manufacture. The right support from pharmaceutical companies, medicines regulatory authorities and national governments is essential.

Achieving vaccine equity presents an essential, but substantial and highly complex, policy challenge. Beyond fundamental issues such as health infrastructure and the availability of trained personnel and medical equipment, unreliable supply and distribution of vaccines in LMICs must be addressed as a matter of urgency, and must happen alongside public health campaigns to challenge misconceptions and address vaccine concerns. Empowering LMICs to develop and/or expand their own vaccine manufacturing capabilities provides a longer-term and more sustainable solution to achieving global vaccination coverage, for COVID-19 and many other infectious diseases. Achieving this necessitates a coordinated effort across multiple agencies, requiring strong national and international leadership and formation of public–private partnerships, as well as scientific and technical expertise and public pressure to instigate change. To surmount this global pandemic, we have a collective responsibility to find a global solution.
